# Genome wide *in vivo* mouse screen data from studies to assess host regulation of metastatic colonisation

**DOI:** 10.1038/sdata.2017.129

**Published:** 2017-09-12

**Authors:** Louise van der Weyden, Natasha A. Karp, Agnieszka Swiatkowska, David J. Adams, Anneliese O. Speak

**Affiliations:** 1 Wellcome Trust Sanger Institute, Wellcome Genome Campus, Hinxton CB10 1SA, UK; 2 Quantitative Biology, IMED, AstraZeneca, Darwin Building (Unit 310), Cambridge Science Park, Milton Road, Cambridge CB4 0WG, UK

**Keywords:** Metastasis, High-throughput screening, Cancer models, Mouse

## Abstract

The process of metastasis is a multi-stage cascade with prior studies suggesting that the colonisation of the secondary site is the rate limiting step. This process involves contributions from the tumour cells and also non-tumour intrinsic factors such as the stroma and the haematopoietic system. In this study, we present data from screening 810 genetically-modified mouse lines with the experimental metastasis assay where intravenous delivery of murine metastatic melanoma B16-F10 cells was used to assess the formation of pulmonary metastasic foci. To date, these data have been studied with a two-step process cumulating in an integrative data analysis to identify genes controlling metastatic colonisation. We present the raw data, and a description to support fresh analyses where researchers can look both within and across gene sets to further elucidate process that regulate metastatic colonisation.

## Background & Summary

Metastasis, the process of tumour cells seeding in secondary sites, is the leading cause of death for cancer patients^1^. This multi-step process first requires the tumour cell to invade into adjacent tissues and then undergo intravasation into the blood stream or lymph, for these cells to travel around the body as a circulating tumour cell (CTC), arresting at a distant site and then extravasate. At this secondary site the tumour cells must establish a niche in order to survive and proliferate at the new site (i.e., ‘colonisation’ of the secondary organ). Research using animal models has determined that the initial steps of the metastatic cascade are relatively efficient and that the rate-limiting step is the process of colonisation at the secondary site, where a tumour cell must survive and proliferate rather than become dormant or be eliminated^2^. Many studies have investigated the process of metastasis with respect to tumour cell intrinsic factors, investigating how mutations and changes within the tumour cell can alter this process^3^, yielding many important insights. Studies utilising cancer-prone mouse models crossed to different genetic backgrounds have identified genetic loci and polymorphisms altering primary tumour formation and metastasic burden for prostate^4^, melanoma^5^ and mammary cancer^6^ models.

Here we sought to identify tumour cell extrinsic factors that can regulate pulmonary metastatic colonisation using high-throughput screening of genetically-modified mouse lines. Taking advantage of the well-established experimental metastasis assay, whereby cells are injected into the bloodstream, thus mimicking CTCs, we tail vein injected B16-F10 mouse melanoma cells into 810 genetically-modified mouse lines. In this way, we aimed to determine the ability of tumour cells to colonise the lung of mutants 10 days after injection, relative to age-, sex- and strain-matched wildtype (control) mice. This assay allows the assessment of the role of host genes in altering survival of CTCs, adherence and extravasation of these cells within the pulmonary capillary bed and their survival and proliferation within the lung microenvironment where they form metastatic foci that can be enumerated. Analysis of these data identified 23 genes that when altered in the mouse result in either increased or decreased pulmonary metastatic colonisation^7^. In our published study^7^ we reported the output of the statistical analysis, however to reduce duplication and support further analysis both within a genetically-modified mouse line and across lines, this data descriptor provides a detailed description of the raw data and statistical output of the original analysis^7^.

## Methods

### Study design

**Figure 1:**
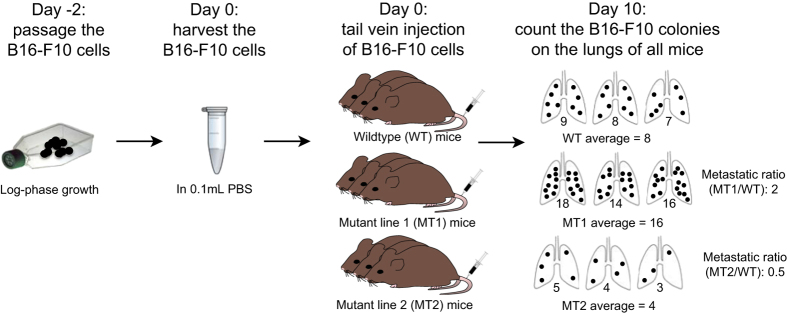
Schematic of the B16-F10 pulmonary metastasis screen. B16-F10 cells are passaged 2 days prior to intravenous administration to ensure they are in log-phase growth when collected. Cells are collected and prepared for administration to wildtype control and mutant mice that are culled 10 days later for counting B16-F10 colonies on the lungs. The total count is determined and for each mutant line (MT1 and MT2) the metastatic ratio is derived by comparing the average of the concurrent wildtype controls and the average of the mutant cohort (see example values given) and significance determined by Mann-Whitney test. Additional cohorts are screened when the Mann-Whitney *P* value was≤0.0175 and the metastatic ratio was≤0.6 or≥1.6, and all the data combined to run through an Integrative Data Analysis.

The aim of the study was to use the experimental metastasis assay in a high-throughput setting such that hundreds of mutant mouse lines could be screened and the methods below are an expanded version of those previously reported^7^. A two-step process was used to identify robust and reproducible effects of the mutant allele on metastatic colonisation. In the first stage, an initial cohort of mice for a mutant allele were studied and the data analysed via a Mann Whitney test as described in the statistical section below with additional cohorts assayed independently for an integrated analysis across cohorts. The workflow within a single experimental run is shown in Fig. 1, the mice were tail vein dosed with a defined number of B16-F10 murine melanoma cells and then 10 days later they were humanely sacrificed and the number of metastatic melanoma lesions (‘colonies’) on the lung counted by visual inspection.

There were no *a priori* estimates of the sample size required and due to the high-throughput nature of the screen the number of mutant mice per cohort varied, however, a typical dosing group consisted of 3–4 mutant lines (with 3–6 mice per mutant line) and 10–20 wildtype mice. Sex-, age- and strain-matched wildtype control mice were always dosed at the same time as the mutant mice. Mutant and wildtype mice were selected across different litters and matings to minimize potential litter and/or cage effects. The wildtype controls were predominantly from a breeding programme of heterozygous x heterozygous or heterozygous x wildtype matings used to generate the mutant of interest (line mate controls). On some occasions, the wildtype controls were from wildtype colonies (production colony controls). In order to minimise false positives in the screen a two-step process was implemented, where the first cohort was screened for potential and if significant, further cohorts were collected. In addition, for a number of alleles which were not significant at the first stage, further cohorts were collected in order to estimate the false negative rate of the screen.

Researchers were not blinded to genotype at any stage of the experiment (as it was written on the cage card), however, as a high-throughput program, bias was reduced as there was no investment in each line, and the researchers had no *a priori* knowledge or assumption as to the experimental outcome for any particular allele. Due to the number of animals processed, body weight was not a parameter that was recorded in this study, however, as the majority of the mutant alleles were subjected to a high-throughput phenotyping pipeline^8^ that included body weight assessment, any phenodeviants for this trait would be identified via this parallel analysis.

### Mice

The majority of mutant mice were generated using targeted embryonic stem (ES) cell clones obtained from the European Conditional Mouse Mutagenesis (EUCOMM) Programme/Knockout Mouse Project (KOMP)-CSD collection^9^. Some alleles were generated using CRISPR/Cas9 techniques either targeting the critical exon (to result in gene deletion) or to create a point mutation^10^. Full details of the particular allele used for each mutant line is provided in Data Record 1.

The majority of mice (>98%) were on the C57BL/6 core strain background (B6) with the specific sub-strains listed in Data Record 1 including strain groups. The mutant mice analysed were predominantly homozygotes for a single mutant allele (67% of the lines tested). Heterozygote mice were analysed if the line was classed as lethal or sub-viable; a classification defined as the scenario where 13% or less of homozygotes were obtained following the genotyping of 28 or more offspring from heterozygous parents. Of the mutant alleles screened 21.7% were lethal, 7.3% subviable and 70.9% classified as viable. A total of 40 genes were screened as both heterozygote and homozygote, 237 genes screened as heterozygote only and 532 genes screened as homozygote only. Mice were typically dosed with tumour cells at 6 to 8 weeks old in the experimental metastasis assay, but due to the nature of the screen occasionally to get a sufficient number of mutant animals this needed to be extended (and in which case, age-matched controls were used). The ages of the mice (on the counting day ‘assay date’) are listed in Data Record 2 and would combine mice of a±2-week age difference for analysis (except in the pilot phase of the study, when a 4-week age difference was tolerated, for the first two months of the reported data).

The care and use of all mice in this study were in accordance with the UK Animals in Science Regulation Unit’s Code of Practice for the Housing and Care of Animals Bred, Supplied or Used for Scientific Purposes, the Animals (Scientific Procedures) Act 1986 Amendment Regulations 2012, and all procedures were performed under a UK Home Office Project licence (PPL 80/2562), which was reviewed and approved by the Sanger Institute’s Animal Welfare and Ethical Review Body. All mice were housed in individually ventilated cages (Techniplast GM500) receiving 60 air changes per hour, in a specific pathogen free environment with ad libitum access to autoclaved water and food (Mouse Breeders Diet, Laboratory Diets, 5,021-3). Cages were filled with aspen bedding substrate, with a nestlet and fun tunnel for environmental enrichment. There was a 12-hour light/dark cycle with no twilight period with a temperature of 21 °C±2 °C and a humidity of 55%±10%. A cage density of one to six mice was used and wildtype and mutant mice were typically in separate cages (unless they were littermates). If a cage of mice were found fighting once they had been dosed (as evidenced by fight wounds on one or more of the mice, typically on the rump), they were solo-housed for the remainder of the study to prevent further fights and the individual that had been bitten was humanely culled (and excluded from the study as they did not reach the required end point).

### Cell line

The C57BL/6-derived mouse melanoma B16-F10 cell line^11^ was purchased from ATCC (CRL-6475) and maintained in DMEM (high-glucose) with 10% (v/v) foetal calf serum, 2 mM glutamine and 100 Uml^−1^ penicillin/streptomycin at 37 °C, 5% CO_2_. The cell line was screened for the presence of mouse pathogens and mycoplasma (Charles River Laboratories MAP test) and validated for the presence of known mutations by whole genome sequencing (accession ERP001691). Prior to injection, the cells (which were cultured for no more than 3–5 passages) were trypsinised, resuspended in media (as above) and counted (BioRad TC20 automated cell counter, average of 6 replicates). The desired number of cells were then centrifuged, and resuspended in phosphate buffered saline (PBS) to the required concentration.

### Pulmonary metastasis screen

Mice were tail vein dosed with 4–5×10^5^ B16-F10 cells as indicated Data Record 2, in a volume of 0.1 ml PBS. The difference in doses was due to the fact that as the screen evolved over time, we noticed that a dose of 5×10^5^ B16-F10 cells made it difficult to count the number of pulmonary colonies in mutant mice with strongly increased pulmonary metastases, so we lowered the dose to 4.5 and then 4×10^5^ cells. At the time of dosing, a note was made if there were any technical issues with the dosing (such as difficulty in locating a vein or mouse moved whilst the needle was in the vein such that it may have been displaced) to use later in quality control of the data (pre-defined criteria listed in Table 1). The same individual performed all dosing to minimise variation due to operator effects. Typically, a maximum of 50 mice were in a single dosing group and wildtype mice were dosed at the start and end of the group. On the occasions when the dosing group was >60 mice, additional wildtype mice were dosed in the ‘middle’ of the group (in case there were significant differences in the number of counts between the wildtypes dosed at the beginning of the session and those dosed at the end of the session).

After ten days (±1 day) all the mice from a cohort were sacrificed via cervical dislocation (on a cage-by cage basis, with no specific order in which the cages were processed), and their lungs removed and rinsed in phosphate buffered saline. The number of B16-F10 colonies on all 5 lobes of the lung were then counted visually and recorded. To reduce variation, all counts of a dosing group were performed by a single individual, who was not blinded to the genotype of the mice. The experiment was designed to minimise welfare concerns, as such with the short time-course of the screen (i.e., 10 days from dosing to collection) the mice did not show any signs of pain, distress or other clinical symptoms due to their pulmonary metastatic burden. Throughout the experiment, the welfare of the mice was monitored with daily visual checks, and if a mouse did display welfare concerns during the 10 days (due to an unrelated issue), it was sacrificed by cervical dislocation and excluded from the study.

### Quality control

The exclusion criteria for the study is listed in Table 1. Individual wildtype (control) mice with a lung colony count of ≤35 were excluded from the analysis with the assumption being that dosing of the full 0.1 ml volume into the tail vein had not been successfully achieved. This was determined on the analysis of hundreds of wildtype mice using the assay. However, low counts obtained from mutant mice were not excluded, as it could not be predicted whether they were due to incomplete dosing or whether this was a result of the mutant allele (i.e., mutation of the gene resulted in decreased pulmonary metastatic colonisation). In contrast, if there were technical difficulties during the dosing procedure that were recorded at the time of dosing regardless of genotype (for example, unable to locate the vein, or if the needle did not remain in the vein whilst the entire dose was administered such as if the mouse moved during the procedure), the data was excluded in the analysis. Where mice had a pulmonary metastasis count of 0 it was assumed that the dosing failed and these were excluded. Where pathology was present at the time of necropsy, such as fight wounds or malocclusion, that resulted in a pulmonary metastasis count that was outlier compared to cage mates this was also excluded from the analysis. All ‘excluded’ data values are blank in Data Record 2 with a comment in QC reason column to indicate the exclusion criteria applied. Out of the 13,426 mice reported in this dataset 4.16% of mutants were excluded and 5.75% of wildtype mice.

### Analysis

Control data was associated with mutant data from the same genetic background, dosing, age and batch. The design of the screen meant that control mice are shared with multiple mutant lines provided that they were dosed in the same cohort. For the purpose of the screen we grouped multiple genetic backgrounds into strain ‘bins’, defined on the basis of pilot studies to investigate if there was an effect on the pulmonary metastasis count. For example, C57BL6/N and C57BL6/J can be combined whereas the presence of the 129 genetic background requires a separate strain bin. Statistical analysis focused on identifying whether there was a statistically significant increase or decrease in pulmonary metastatic colonisation relative to wildtype controls for each mutant line independently. The experimental unit was considered the individual mouse. To allow comparison of mutant lines assayed on different days, a ‘metastatic ratio’ was used (Fig. 1).

### Statistical analysis implemented

Due to the small sample size within the first cohort for a given mutant allele, a Mann-Whitney test was used to compare the mutants to the concurrent wildtype controls dosed in the same cohort and the ‘metastatic ratio’ calculated (whereby the average colonies of a mutant mice line was divided by the average colonies in the wildtype control mice of the same cohort; Fig. 1). A Mann-Whitney *P* value of≤0.0175 with a metastatic ratio of≤0.6 or≥1.6 was chosen as the cut off for the initial phase of selecting phenodeviant lines for further screening. This cut off was chosen allowing a false discovery rate of 15% to manage the false negative rate at this stage of the screen. At least three additional cohorts were then screened ensuring both sexes were assayed (wherever possible) and the data from all cohorts processed through an integrative data analysis to determine those lines that were statistically significant result after controlling the family wise error rate to 5%.

An Integrative Data Analysis (IDA, also called Mega-Analysis)^12^ was completed using R (package nlme v3.1) treating each experiment as a fixed effect. An iterative top down modelling strategy was implemented starting with the most comprehensive model (either Eq. [1] or [2]) appropriate for the collection strategy implemented and ensuring the model only included terms where the terms could be independently assessed.

(1)Y=β0+β1Sex+β2Experiment+β3Genotype+β4SexGenotype

(2)Y=β0+β2Experiment+β3Genotype

The optimisation process first selected a covariance structure for the residual, then the model was reduced by removing non-significant fixed effects, and finally the genotype effect was tested and model diagnostics visualised. For the hypothesis test of primary interest, the impact of genotype, the per-comparison error rate threshold *P* values were adjusted to account for the multiple comparisons to control the family wise error rate to 5% using the Hochberg method^13^. Demonstration of the IDA methodology (Supplementary File 1) and an example dataset (Supplementary File 2) is provided.

## Data Records

The associated data described in the manuscript are available for download from Figshare (Data Citation 1).

### Data Record 1: Complete listing of the mutant mouse genes screened and the associated allele information

A subset of genes (29) were screened as 2 different alleles and for 1 gene a total of 3 alleles have been screened. The different alleles were generally tm1a and tm1b to discern if there was any difference after cre-mediated excision of the critical exon and targeting cassette (for details of the EUCOMM/KOMP-CSD tm1a/tm1b allele nomenclature refer to Skarnes *et al.*^9^). In some instances, one allele was a point mutation and the other targeting the whole gene. ‘Screened gene’ is the gene name as it appears in Data Record 2, followed by the ‘full allele name’, ‘allele superscript’ and ‘allele MGI ID’ (where a record exists). The ‘unique identifier’ is the internal colony prefix that was used for tracking and is specific to the gene and allele within the institute. In some instances, there are multiple colony prefixes for the same gene and allele—this occurred when additional mice were generated for testing outside of the high-throughput screening project. ‘MGI Gene/Marker ID’ allows tracking of the gene independent of any change in gene name for which a number have changed. The ‘current symbol’ is the most recent gene name and ‘synonym screened’ is where the gene has since been changed at MGI (and also internally) so allows for cross checking to the original data. The full current gene name and feature annotation is listed together with the chromosomal location of the gene and the Ensembl ID.

### Data Record 2: Complete data set for all the mice comprising the 810 genes screened

Results of the B16-F10 pulmonary metastasis screen for the 810 genes consisting of 13,426 individual mice.

## Technical Validation

**Figure 2:**
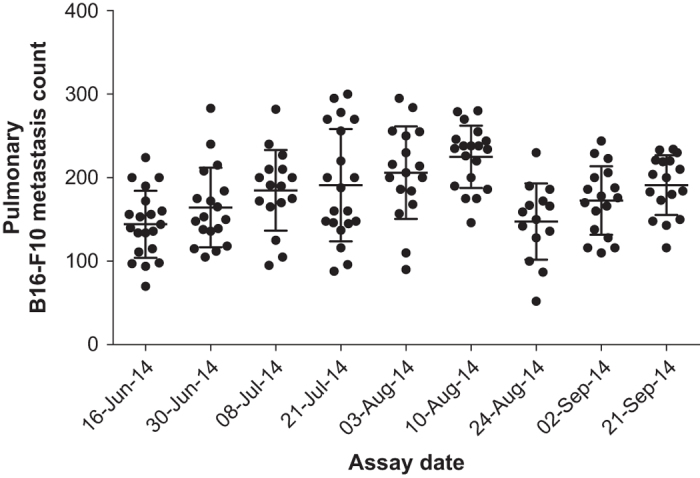
Temporal variation in metastasis data. B16-F10 pulmonary metastasis count values from female wildtype controls of the B6 strain group administered over time (horizontal line at mean with error bar standard deviation, each symbol represents an individual mouse). As there is batch-to-batch variation, mutant values can only be compared to the concurrent controls administered the same batch of cells.

At the start of the study, the selected dose was 5×10^5^ B16-F10 cells based on reading scientific literature (B16-F10 cells have been widely used since their creation in the 1970s (ref. 11)). Pilot experiments were performed involving mutant mice known to exhibit decreased metastatic colonisation (*Cybb*^*tm1Din*^ (ref. 14)) and increased metastatic colonisation (*Irf1*^*tm1a(EUCOMM)Wtsi*^; other *Irf1* alleles were demonstrated to have severely impaired natural killer cells^15^ and a CD8 T cell defect^16^—key immune system regulators of metastasis), as positive controls to validate the screen. Further pilot studies, demonstrated that sex, age and genetic background could confound the experiment. Therefore, the experiment was designed to account for these sources of variation by standardisation and only associating control data with equivalent meta-data to the mutant mice to avoid these effects confounding the experiment. Temporal variation (Fig. 2a) is to be expected^17^ and is why concurrent controls were run throughout the study. No evidence was seen for drift, or seasonal effects within the control data with time. In addition, the ‘metastatic ratio’ was reproducible between multiple experiments for the same gene (Table 2).

To test the false discovery rate of the first step selection, additional cohorts were assayed for 102 mutant lines that were not significant at stage 1 (Mann Whitney *P*>0.0175), and none of these were significant by integrative data analysis (*P*>0.01). This indicates the selection criteria at stage 1 was appropriate and avoided a significant issue with false negatives but significantly increased the throughput allowing stage 2 additional testing to focus on potential lines of interest. The selection criteria for stage 1 lead to 39 significant lines for further study at an estimated false discovery rate (FDR) of 5%. At stage two testing, 59% were still significant when using the more conservative family wise error rate (FWER) of 5%.

## Usage Notes

For the raw data (Data Record 2), there are a number of meta-data (such as sex, genetic background, assay date, zygosity, gene, QC notes and cell number used) that should be considered in any analysis. Table 3 (available online only) details each column of the data explaining the data format, data options and any other relevant information. As this list contains additional data compared to the original manuscript, when more cohorts for genes have been screened, the values from the IDA will be subtly different to those initially reported^7^.

## Additional Information

**How to cite this article:** van der Weyden, L. *et al.* Genome wide *in vivo* mouse screen data from studies to assess host regulation of metastatic colonisation. *Sci. Data* 4:170129 doi: 10.1038/sdata.2017.129 (2017).

**Publisher’s note:** Springer Nature remains neutral with regard to jurisdictional claims in published maps and institutional affiliations.

## Supplementary Material



Supplementary File 1

Supplementary File 2

## Figures and Tables

**Table 1:** Exclusion criteria applies to screen data.

**Exclusion categories for mice**	**Wildtypes**	**Mutants**	**Comment**
Excluded (difficulty dosing)	x	x	This is when a comment was made at the time of dosing that there were some difficulties in ensuring the full dose (0.1 ml) was administered into the vein
Excluded (count=0 so presumed failed dosing)	x	x	This is when there were no mets present, and we know that is impossible for a WT (and highly improbable for a mutant)
Excluded (count ≤35 so presumed failed dosing)	x		This is when there were less than or equal to 35 mets present, and we know from historical WT data that indicates that the full dose was not administered
Excluded (pathology present at time of necropsy)	x	x	This is when the mouse was found to have some abnormality at the time of necropsy (not present or observable at the time of dosing) that may have an impact on the met count (examples include malocclusion, enlargment of an internal organ, scratching, fight wounds)
The exclusion criteria, genotype group it can be applied to and reasoning is indicated.			

**Table 2:** Example of Metastatic ratio stability.

**Gene**	**Sex**	**Assay date**	**Cell number**	**N Mut**	**N WT**	**MR**
*Irf1*	Female	31/08/2012	5×10^5^	3	6	2.39
*Irf1*	Female	08/10/2012	5×10^5^	5	10	2.23
*Irf1*	Male	08/10/2012	5×10^5^	4	7	3.04
*Irf1*	Female	31/05/2012	4×10^5^	3	19	3.85
*Rspo4*	Female	25/01/2015	4×10^5^	6	18	1.08
*Rspo4*	Female	03/03/2015	4×10^5^	4	20	0.90
*Rspo4*	Male	03/03/2015	4×10^5^	4	13	1.05
*Rspo4*	Male	09/03/2015	4×10^5^	5	19	0.93
The metastatic ratio (MR) is shown for two example genes across multiple cohorts.						

**Table 3:** Headings and explanation of Data Record 2 format

**Mouse Name**	**Colony Prefix**	**Genotype**	**Zygosity**	**Sex**	**Birth Date**	**Gene Name**	**Assay Date**	**Age (weeks)**	**Pulmonary Metastasis Count**	**Core Strain**	**Cell Number Administered (×10**^**5**^)	**Dosing Group**	**Full Strain**	**Cell Line Used**	**QC reason**
*Unique mouse identifier*	*Unique identifier for the line—can cross reference the gene list table to look up*	*Full genotype*	*Genotype classification—options are wildtype, heterozygote or homozygote (for X linked genes for ease of filtering hemizygote has been set to homozygote)*	*Sex of the mouse—Female or Male*	*Date of birth for the mouse*	*For a mutant mouse the gene that was targeted*	*Date pulmonary metastasis count was performed*	*Age of the mouse in weeks on the assay date—only mice with the same assay date can be compared +/− 2 weeks*	*Count value, blank if data have been QC failed*	*These are the strain groups of mice that can be combined from a strain perspective*	*Dose of cells used*	*These are the cohorts of mice that were administered with the same preparation of cells. Due to subtle differences with each batch of cells only mice within the same dosing group should be compared*	*The full genetic background of the mouse*	*Cell line used in the assay*	*Reason for excluding data as outlined in Table 1*
CBLT1300.4d	CBLT	+/+	Wildtype	Female	25-May-12		13-Aug-12	11.4	123	B6	5	A	C57BL/6NTac	B16-F10	
			*Options*	*Options*						*Options*	*Options*	*Options*	*Options*	*Options*	
			Wildtype	Female						B6	4	A	C57BL/6NTac	B16-F10	
			Heterozygous	Male						B6JIco;B6J;129P2;B6Brd	4.5	B	C57BL/6J;Stock		
			Homozygous							B6JIco;129P2	5	C	C57BL/6Brd-Tyr<c-Brd>(37.5%);C57BL/6Dnk(25%);C57BL/6N(37.5%)		
										B6JIco;B6Brd;129S5-Tyr<c-Brd>		D	C57BL/6N;C57BL/6NTac		
										129/SvEv		E	C57BL/6N(50%);C57BL/6NTac(50%)		
										STOCK Cdh23<v>			C57BL/6N(37.5%);C57BL/6NTac(62.5%)		
										B6JIco;B6Brd;129P2-Tyr<c-Brd>			C57BL/6N(7.03125%);C57BL/6NTac(92.96875%)		
													C57BL/6N(6.25%);C57BL/6NTac(93.75%)		
													C57BL/6J		
													C57BL/6Brd-Tyr<c-Brd>(12.5%);C57BL/6Dnk(25%);C57BL/6N(12.5%);C57BL/6NTac(50%)		
													C57BL/6N(20.3125%);C57BL/6NTac(79.6875%)		
													C57BL/6Brd-Tyr<c-Brd>;C57BL/6N;C57BL/6NTac		
													C57BL/6Brd-Tyr<c-Brd>(32.8125%);C57BL/6Dnk(9.375%);C57BL/6N(32.8125%);C57BL/6NTac(25%)		
													C57BL/6N(9.375%);C57BL/6NTac(90.625%)		
													C57BL/6N(12.5%);C57BL/6NTac(87.5%)		
													C57BL/6N(25%);C57BL/6NTac(75%)		
													C57BL/6Brd-Tyr<c-Brd>(50%);C57BL/6N(50%)		
													C57BL/6Dnk;C57BL/6NTac		
													C57BL/6Brd-Tyr<c-Brd>;C57BL/6Dnk;C57BL/6NTac		
													C57BL/6Brd-Tyr<c-Brd>;C57BL/6Dnk;C57BL/6N;C57BL/6NTac		
													C57BL/6JIco;C57BL/6Brd-Tyr<c-Brd>;129S5/SvEvBrd/Wtsi;C57BL/6J		
													C57BL/6Brd-Tyr<c-Brd>(34.960938%);C57BL/6Dnk(30.078125%);C57BL/6N(34.960938%)		
													C57BL/6Brd-Tyr<c-Brd>;C57BL/6Dnk;C57BL/6NTac;C57BL/6		
													C57BL/6N(47.65625%);C57BL/6NTac(52.34375%)		
													C57BL/6N(49.21875%);C57BL/6NTac(50.78125%)		
													C57BL/6N(48.4375%);C57BL/6NTac(51.5625%)		
													C57BL/6Dnk		
													C57BL/6JIco;129P2/OlaHsd		
													C57BL/6JIco;C57BL/6Brd-Tyr<c-Brd>;129S5/SvEvBrd/Wtsi		
													C57BL/6N		
													C57BL/6J(100%)		
													C57BL/6Brd-Tyr<c-Brd>;C57BL/6Dnk;C57BL/6N		
													C57BL/6NTac(100%)		
													C57BL/6N(21.875%);C57BL/6NTac(78.125%)		
													C57BL/6NTac;Stock		
													129S5/SvEvBrdWtsi(100%)		
													Stock(100%)		
													C57BL/6JIco;C57BL/6Brd-Tyr<c-Brd>;129P2/OlaHsdWtsi		
The formatting of Data Record 2 is explained together with a list of all the possible options for some of the key columns.															
