# Supplementary material for: Genome wide in vivo mouse screen data from studies to assess host regulation of metastatic colonisation
Source: Sci Data. 2017 Sep 12;4:170129. doi: 10.1038/sdata.2017.129 (PMC5827107; doi:10.1038/sdata.2017.129)
Supplement: Supplementary File 1 [file sdata2017129-s2.docx]

Demonstration of the Integrated Data Analysis

Natasha Karp

# Overview

Ths document demonstrates an Integrated Data Analysis for a dataset.

## Methodology overview:

Integrative Data Analysis (also known as Mega-Analysis) was completed using R (package nlme v3.1) treating each experiment as a fixed effect. An iterative top down modelling strategy was implemented starting with the most comprehensive model (either Eq. [1], or [2]) appropriate for the collection strategy implemented and ensuring the model only included term where the terms could be independently assessed.

Y ~ Sex + Experiment + Genotype +Sex*Genotype [Eq.1]

Y ~ Experiment + Genotype [Eq.2]

The optimisation process first selected a covariance structure for the residual, then the model was reduced by removing non-significant fixed effects, and finally the genotype effect was tested and model diagnostics visualised. For the hypothesis test of primary interest, the impact of genotype, the per-comparison error rate threshold P values were adjusted to account for the multiple comparisons to control the false discovery rate to 5% using the Hochberg method.

Note: Within this script only one dataset is processed; so no multiple testing control is implemented. Results would need combining across datasets for this adjustment.

# Inputting the data for analysis and graphing

The analysis requires a dataframe with one header row and one row for each animal.

The analysis requires the following columns

1. genotype - with two strings to represent the testGenotype and refGenotype (e.g. WT and HOM)
2. sex - with two strings (M/F) to represent Male and Female
3. experiment - a column with strings to represent the batches in which the data was collected.
4. outcome - a column of the number of pulmonary metastasis counted.

setwd("C:\\Users\\kppq436\\OneDrive - AZCollaboration\\Louise WH")
data=read.csv("Arhgef1.csv")
data=data[data$sex!="", ]
data$genotype=as.factor(data$genotype)

graphBySexGenotypeExperiment<-function(df){

 Male <- subset(df, df$sex=="M")
 Female <- subset(df, df$sex=="F")
 op <- par(mfrow=c(1,2))
 y_range <- c(min(df[ ,"outcome"], na.rm=TRUE), max((df[ ,"outcome"]), na.rm=TRUE))
 stripchart(Male[ , "outcome"]~Male$experiment, pch=1,vertical=TRUE,
 subset=(Male$genotype=="+/+"), ylim=y_range,xlab="Experiment", ylab="Number metastases", cex.lab=1.4)
 points(Male[ , "outcome"]~Male$experiment, subset=(Male$genotype!="+/+"), col="red")
 legend("topleft",c("WT","KO"), pch=c(1), bty="n", col=c("black","red"))
 legend("top", "Male", cex=1.3, bty="n")
 stripchart(Female[ , "outcome"]~Female$experiment, pch=1,vertical=TRUE,
 subset=(Female$genotype=="+/+"), ylim=y_range, xlab="Experiment", ylab="Number metastases", cex.lab=1.4)
 points(Female[ , "outcome"]~Female$experiment, subset=(Female$genotype!="+/+"), col="red")
 legend("top", "Female", cex=1.3, bty="n")
 legend("topleft", c("WT","KO"), pch=c(1), bty="n", col=c("black","red"))
 par(op)
 op_normal <- par(mfrow=c(1,1))
 par(op_normal)

}

graphBySexGenotypeExperiment(data)


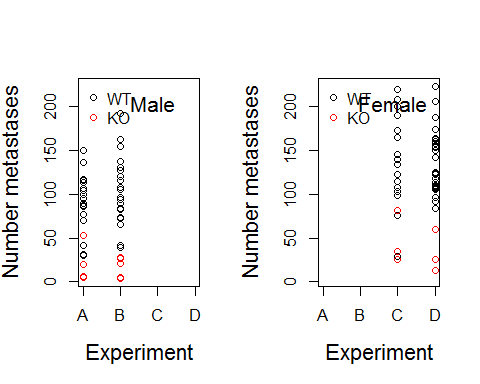


# Assessing the design of the experiment

To select the appropriate model for statistical analysis, the following code asseses whether male and female data was collected on the same day. Note the code assumes that everyday data is collected has both wildtype and knockout data - which is the design so should be safe assumption.

# a function that counts data points for a column of a dataframe
countDataPoints<-function(dataset, depVariable){
 return(sum(is.finite(dataset[ , depVariable])))
}

output_counting=ddply(.data=data, .variables=c("experiment","sex", "genotype" ), .fun=countDataPoints, depVariable="outcome")
KOcountDataonly=output_counting[output_counting$genotype!="+/+",]

bothSexStudiedSameExperiment=length(unique(KOcountDataonly$experiment)) != nrow(KOcountDataonly)
print(c("Did this experiment have both sexes studied within a single experiment?", bothSexStudiedSameExperiment))

FALSE [1] "Did this experiment have both sexes studied within a single experiment?"
FALSE [2] "FALSE"

# Fit the statistical model and assess the model diagnostics

The table of results tells you the impact of the knockout and the experiments on the measurement.

There are two plots to assess the quality of the estimate from the statistical model

1. The Q-Q plot tells you the distribution of the residuals (the difference between the actual measurement and that predicted from the model). this statistical model is assuming the residuals will be normally distributed. This can be assessed with the QQ plot which is is assessing the distribution of the residuals against a normal distribution. If both sets of quantiles came from the same distribution, we should see the points forming a line that's roughly straight.
2. The second plot is used to visually assess for patterns in the residuals (the difference between the actual measurement and that predicted from the model) by plotting then against the predicted. We would be concerned if the residuals had a systematic pattern depending on the predicted signal strength.

Finally a vector of output is generated using data from the table to allow easy comparison across multiple datasets.

#A function to return the starting formula to fit. Depends on the modeltype (test/null) and whether there is data from both sexes collected on the same day (bothSexStudiedSameExperiment =TRUE/FALSE)

model_Formula <- function(modelType, DidExperimentbothSexStudied){
 if(DidExperimentbothSexStudied==TRUE){
 if(modelType=="test"){
 model.formula <- as.formula(paste("outcome", paste("genotype", "experiment", "sex", "genotype*sex", sep= "+"), sep="~"))
 }else{
 model.formula <- as.formula(paste("outcome",paste("experiment", "sex", sep= "+"), sep="~"))
 }
 }else{
 if(modelType=="test"){
 model.formula <- as.formula(paste("outcome", paste("genotype", "experiment", sep= "+"), sep="~"))
 }else{
 model.formula <- as.formula(paste("outcome", "experiment", sep= "~"))
 }
 }
 return(model.formula)
}

#A function to return the finally fitted model for estimation of genotype effect. Three arguments for input keepSex: TRUE or FALSE, keepInteraction: TRUE or FALSE and type: test or null.

simplifiedModel<-function(keepSex, keepInteraction, type){
 if(type=="test"){
 if (!keepSex && !keepInteraction){
 model.formula <- as.formula(paste("outcome", "~", paste("genotype", "experiment", sep= "+")))
 }else if((!keepSex && keepInteraction)|(keepSex && keepInteraction)){
 model.formula <- as.formula(paste("outcome", paste("genotype:sex","experiment", "sex", sep= "+"), sep="~"))
 }else if(keepSex && !keepInteraction){
 model.formula <- as.formula(paste("outcome", "~", paste("genotype", "experiment", "sex", sep= "+")))
 }
 }else{
 if (!keepSex && !keepInteraction){
 model.formula <- as.formula(paste("outcome", "~", "experiment"))
 }else if((!keepSex && keepInteraction)|(keepSex && keepInteraction)){
 model.formula <- as.formula(paste("outcome", paste("experiment", "sex", sep= "+"), sep="~"))
 }else if(keepSex && !keepInteraction){
 model.formula <- as.formula(paste("outcome", "~", paste("experiment", "sex", sep= "+")))
 }
 }
 return(model.formula)
}

#A function to return the residuals from the final model fitted plotted as a function of genotype group. Input is a dataframe and the model output following model fitting.

ResidualQQplot_bygenotype<-function(df, model){
 res=resid(model)
 data_all= data.frame(df, res)
 a=levels(data_all$genotype)
 par(mfrow = c(1, 2))
 Gp1 = subset(data_all, genotype==a[1])
 Gp2 = subset(data_all, genotype==a[2])
 qqnorm(Gp1$res, main=" ")
 qqline(Gp1$res)
 legend("topleft", a[1], cex=1.3, bty="n")
 close.screen(n=1, all.screens = FALSE)
 qqnorm(Gp2$res, main=" ")
 qqline(Gp2$res)
 legend("topleft", a[2], cex=1.3, bty="n")
}

#A function to assess whether the residuals distribution depends on signal. Input is a dataframe and the model output following model fitting.

ResVpred_bygenotype<-function(df,model){

 pred = predict(model)
 res=resid(model)
 data_All= data.frame(df, res, pred)
 a=levels(df$genotype)
 genotype_no=length(a)
 par(mfrow = c(1, 2))
 Gp1pred = subset(data_All, genotype==a[1])
 Gp2pred = subset(data_All, genotype==a[2])
 plot(x=Gp1pred$pred, y=Gp1pred$res, xlab="Predicted", ylab="Residual")
 legend("topleft", a[1], cex=1.3, bty="n")
 plot(x=Gp2pred$pred, y=Gp2pred$res, xlab="Predicted", ylab="Residual")
 legend("topleft", a[2], cex=1.3, bty="n")
}

#A function to fit the model and return the diagnostics and output values for a dataframe Input is a dataframe and whether within this dataset there was data collected from both sexes on the same data (wereBothSexesStudiedSameExperiment options: TRUE/FALSE).

megaAnalysisModelFitting<-function(df, wereBothSexesStudiedSameExperiment){

 #first step the covariance structure ie can we assume eqaul variance across the genotype group
 testModel=model_Formula(modelType="test", wereBothSexesStudiedSameExperiment)
 nullModel=model_Formula(modelType="null", wereBothSexesStudiedSameExperiment)
 model_test_homV <- do.call("gls", args=list(testModel, df,method="ML", na.action="na.omit"))
 model_test_hetV <- do.call("gls", args=list(testModel, df,method="ML", na.action="na.omit", weights=varIdent(form=~1|genotype)))
 EqualVar=anova(model_test_homV, model_test_hetV)$`p-value`[2]>0.05

#to know where to find information in table - note as it will be compared to the reference (experiment a) - 3 separates batches will add two rows to the table
 noExperiments=length(levels(df$experiment))

 if(EqualVar=="TRUE"){

 if(wereBothSexesStudiedSameExperiment==FALSE){

 Final_model= model_test_homV
 null_model= do.call("gls", args = list(nullModel, data = df, na.action="na.exclude",method="ML"))
 pvalue=anova(Final_model, null_model)$p[2]
 Final_model= do.call("gls", args = list(testModel, data = df,na.action="na.exclude", method="REML"))
 ModelResults=(summary(Final_model)$tTable)
 print(ModelResults)
 print(c("testing role of genotype via IDA=", pvalue))
 ResidualQQplot_bygenotype(df, model=Final_model)
 ResVpred_bygenotype(df, model=Final_model)
 output=c(pvalue, ModelResults[2], ModelResults[4+(noExperiments-1)], NA, NA, NA, NA)
 names(output)=c("pvalue", "Genotype_estimate", "Standard Error", "Genotype*Female Estimate", "Genotype*Male Estimate", "Genotype*Male SE", "Genotype*Female SE")
 print(output)


 }else{

 anova_results = anova(model_test_homV, type="marginal")$"p-value" < 0.05 #1st: intercept, 2nd: Genotype, 3: Experiment, 4: Sex, 5: Genotype*sex
 keepSex= anova_results[4]
 keepInteraction= anova_results[5]

 finalModelFormula=simplifiedModel(keepSex, keepInteraction, type="test")
 nullModelFormula=simplifiedModel(keepSex, keepInteraction, type="null")
 null_model= do.call("gls", args = list(nullModelFormula, data = df, na.action="na.exclude",method="ML"))
 final_model= do.call("gls", args = list(finalModelFormula, data = df, na.action="na.exclude",method="ML"))
 pvalue=anova(final_model, null_model)$p[2]
 Final_model= do.call("gls", args = list(finalModelFormula, data = df,na.action="na.exclude", method="REML"))
 ModelResults=(summary(Final_model)$tTable)
 print(ModelResults)
 print(c("testing role of genotype via IDA=", pvalue))
 ResidualQQplot_bygenotype(df, model=Final_model)
 ResVpred_bygenotype(df, model=Final_model)

 if((!keepSex && keepInteraction)|(keepSex && keepInteraction)){

 output=c(pvalue, NA, NA, ModelResults[7], ModelResults[8], ModelResults[7+(2*(noExperiments-1))], ModelResults[8+(2*(noExperiments-1))])
 names(output)=c("pvalue", "Genotype_estimate", "Standard Error", "Genotype*Female Estimate", "Genotype*Male Estimate", "Genotype*Male SE", "Genotype*Female SE")

 }else if (!keepSex && !keepInteraction){

 output=c(pvalue, ModelResults[2], ModelResults[4+(noExperiments-1)], NA, NA, NA, NA)
 names(output)=c("pvalue", "Genotype_estimate", "Standard Error","Genotype*Female Estimate", "Genotype*Male Estimate", "Genotype*Male SE", "Genotype*Female SE")

 }else if(keepSex && !keepInteraction){

 output=c(pvalue, ModelResults[2], ModelResults[5+(noExperiments-1)], NA, NA, NA, NA)
 names(output)=c("pvalue", "Genotype_estimate", "Standard Error", "Genotype*Female Estimate", "Genotype*Male Estimate", "Genotype*Male SE", "Genotype*Female SE")
 }

 print(output)

 }


 }else{
 if(wereBothSexesStudiedSameExperiment==FALSE){

 Final_model= model_test_hetV
 null_model= do.call("gls", args = list(nullModel, data = df, na.action="na.exclude", weights=varIdent(form=~1|genotype), method="ML"))
 pvalue=anova(Final_model, null_model)$p[2]
 Final_model= do.call("gls", args = list(testModel, data = df, na.action="na.exclude", weights=varIdent(form=~1|genotype), method="REML"))
 model_test_hetV
 ModelResults=(summary(Final_model)$tTable)
 print(ModelResults)
 print(c("testing role of genotype via IDA=", pvalue))
 ResidualQQplot_bygenotype(df, model=Final_model)
 ResVpred_bygenotype(df, model=Final_model)
 output=c(pvalue, ModelResults[2], ModelResults[4+(noExperiments-1)], NA, NA,NA, NA)
 names(output)=c("pvalue", "Genotype_estimate", "Standard Error", "Genotype*Female Estimate", "Genotype*Male Estimate", "Genotype*Male SE", "Genotype*Female SE")
 print(output)

 }else{

 anova_results = anova(model_test_hetV, type="marginal")$"p-value" < 0.05 #1st: intercept, 2nd: Genotype, 3: Experiment, 4: Sex, 5: Genotype*sex
 keepSex= anova_results[4]
 keepInteraction= anova_results[5]
 finalModelFormula=simplifiedModel(keepSex, keepInteraction, type="test")
 nullModelFormula=simplifiedModel(keepSex, keepInteraction, type="null")
 null_model= do.call("gls", args=list(nullModelFormula, data = df, na.action="na.exclude",weights=varIdent(form=~1|genotype),method="ML"))
 final_model= do.call("gls", args=list(finalModelFormula, data = df, na.action="na.exclude", weights=varIdent(form=~1|genotype), method="ML"))
 pvalue=anova(final_model, null_model)$p[2]
 Final_model= do.call("gls", args = list(finalModelFormula, data = df,na.action="na.exclude", weights=varIdent(form=~1|genotype), method="REML"))
 ModelResults=(summary(Final_model)$tTable)
 print(ModelResults)
 print(c("testing role of genotype via IDA=", pvalue))
 ResidualQQplot_bygenotype(df, model=Final_model)
 ResVpred_bygenotype(df, model=Final_model)

 if((!keepSex && keepInteraction)|(keepSex && keepInteraction)){

 output=c(pvalue, NA, NA, ModelResults[7], ModelResults[8], ModelResults[7+(2*(noExperiments-1))], ModelResults[8+(2*(noExperiments-1))])
 names(output)=c("pvalue", "Genotype_estimate", "Standard Error", "Genotype*Female Estimate", "Genotype*Male Estimate", "Genotype*Male SE", "Genotype*Female SE")

 }else if (!keepSex && !keepInteraction){

 output=c(pvalue, ModelResults[2], ModelResults[4+(noExperiments-1)], NA, NA, NA, NA)
 names(output)=c("pvalue", "Genotype_estimate", "Standard Error","Genotype*Female Estimate", "Genotype*Male Estimate", "Genotype*Male SE", "Genotype*Female SE")

 }else if(keepSex && !keepInteraction){

 output=c(pvalue, ModelResults[2], ModelResults[5+ (noExperiments-1)], NA, NA, NA, NA)
 names(output)=c("pvalue", "Genotype_estimate", "Standard Error", "Genotype*Female Estimate", "Genotype*Male Estimate", "Genotype*Male SE", "Genotype*Female SE")
 }

 print(output)

 }
 }
}


megaAnalysisModelFitting(data, bothSexStudiedSameExperiment)

FALSE Value Std.Error t-value p-value
FALSE (Intercept) 96.749402 7.558998 12.7992364 1.237025e-22
FALSE genotypeArhgef1/Arhgef1 -86.559114 7.117480 -12.1614832 2.726125e-21
FALSE experimentB 6.816337 9.533067 0.7150203 4.762964e-01
FALSE experimentC 39.706051 10.202583 3.8917644 1.815730e-04
FALSE experimentD 33.981950 9.592682 3.5424868 6.088061e-04
FALSE [1] "testing role of genotype via IDA=" "5.09414883547293e-11"


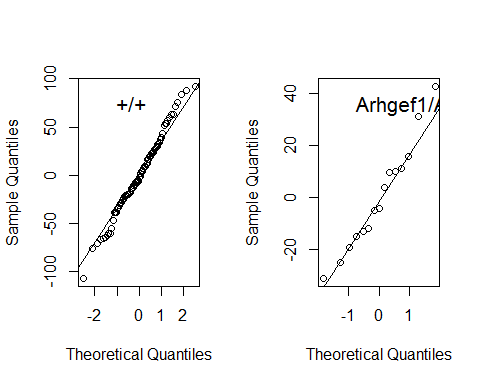

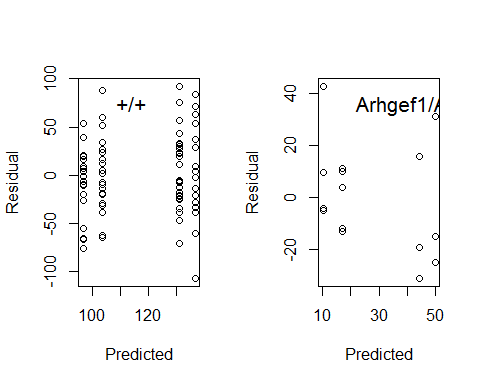


FALSE pvalue Genotype_estimate Standard Error
FALSE 5.094149e-11 -8.655911e+01 7.117480e+00
FALSE Genotype*Female Estimate Genotype*Male Estimate Genotype*Male SE
FALSE NA NA NA
FALSE Genotype*Female SE
FALSE NA
